# International Observatory on Mental Health Systems: a mental health research and development network

**DOI:** 10.1186/1752-4458-3-2

**Published:** 2009-01-22

**Authors:** Harry Minas

**Affiliations:** 1 Centre for International Mental Health, Melbourne School of Population Health, The University of Melbourne, Parkville, Victoria 3010, Australia

## Abstract

**Background:**

While the mental health situation for most people in low and middle-income countries is unsatisfactory, there is a renewed commitment to focus attention on the mental health of populations and on the scaling up of mental health services that have the capacity to respond to mental health service needs. There is general agreement that scaling up activities must be evidence-based and that the effectiveness of such activities must be evaluated. If these requirements are to be realised it will be essential to strengthen capacity in countries to conduct rigorous monitoring and evaluation of system development projects and to demonstrate sustained benefit to populations.

**The Observatory:**

The International Observatory on Mental Health Systems (IOMHS) will build capacity to measure and to track mental health system performance in participating countries at national and sub-national (provincial and district) levels. The work of IOMHS will depend on the establishment of robust partnerships among the key stakeholder groups. The Observatory will build the capability of partner organisations and networks to provide evidence-based advice to policy makers, service planners and implementers, and will monitor the progress of mental health service scaling up activities.

**Summary:**

The International Observatory on Mental Health Systems will be a mental health research and development network that will monitor and evaluate mental health system performance in low and middle-income countries.

## Background

Health systems in low and middle-income countries are characterised by massive under-investment in mental health [1]. The consequences of this are many. There is an almost total reliance on mental hospitals, where quality of treatment and care is generally poor, and there are very few community mental health services [2]. In many places there is a serious shortage of skilled mental health professionals [1] and lack of legislative protections. Poor facilities and lack of skilled mental health workers too often results in neglect and abuse of the human rights of people with mental illness and their families [3].

While effective mental health services are unavailable for most people in low and middle-income countries there is a renewed commitment to focus attention on the mental health of populations and on the scaling up of mental health services that have the capacity to respond to mental health service needs [4]. In October 2008 the World Health Organization Mental Health Gap Action Programme (mhGAP) [5], and the Movement for Global Mental Health [6] were launched. The intent of mhGAP is to scale up care for mental, neurological, and substance use disorders, with strategies identified particularly for resource-constrained settings and countries. The Movement for Global Mental Health (MGMH) aims to improve services for people with mental disorders worldwide through the coordinated action of a global network of individuals and institutions [7].

Mental health system development is also finally making its way onto the agendas of bilateral development agencies and international development NGOs. AusAID is supporting the National Taskforce on Mental Health System Development in Indonesia [4] and other mental health projects and has recently launched an exciting new approach to disability-inclusive development [8], which aims to make disability a development priority. This disability strategy will open the way for substantial and sustained attention to mental disorders, the single most important contributor to disability in low and middle-income countries.

**Figure 1:**
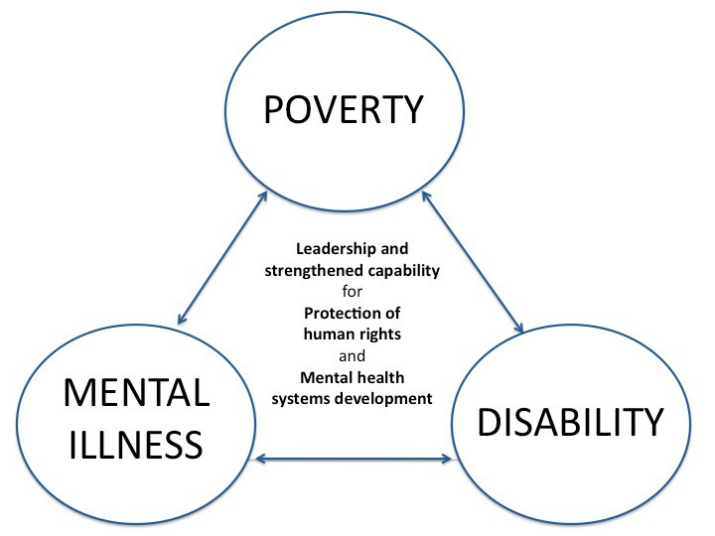
**Poverty, mental illness and disability**.

There is a clear relationship between poverty, mental illness and disability, with the presence of any one factor increasing the likelihood of the others [9-13]. Reducing mental illness and disability, and the poverty that is so commonly a consequence, requires strengthening of human rights protections and development of mental health systems that ensure equitable access to skilled treatment, rehabilitation, social support, housing and employment (Figure 1).

There is general agreement that scaling up activities must be evidence-based and that the effectiveness of such activities must be evaluated. If these requirements are to be realised it will be essential to strengthen capacity in countries to conduct rigorous monitoring and evaluation of system development projects and to demonstrate sustained benefit to populations. Failure to sustain long-term gains from even well designed and implemented community mental health system development projects is a source of serious concern and is all too common. In a follow-up of one such project [14], of the 185 patients followed up, 15% had continued treatment, 35% had stopped treatment, 21% had died, 12% had wandered away from home and 17% were untraceable. Of the patients who had discontinued treatment 25% were asymptomatic while 75% were acutely psychotic. The lessons learned from such a failure of sustainability must be widely disseminated and incorporated into planning of future development projects.

The WHO Mental Health Gap Action Programme [5] identifies examples of indicators (Table 1) that can be used for purposes of monitoring and evaluation. WHO recognises that each country will need to decide which indicators to measure and for what purpose; when and where to measure them; how to measure them; and which data sources to use. Countries will also need to plan for analysis and use of the data. The possible sources of data include reports from health authorities and facilities, auditing of health facilities, national or district programme records, health facility or provider surveys, household surveys, and studies designed to investigate specific issues.

**Table 1:** WHO Mental Health Gap Action Programme – examples of indicators

**Health System**		
**Inputs**	**Outputs**	**Outcomes**

• the number of sites in the country that implement the scaling up strategy;	• the proportion of facilities for primary health that have trained health professionals for diagnosis and treatment of MNS disorders; and	• the number of people treated each year for MNS disorders as a proportion of the total estimated yearly prevalence of MNS disorders.
• the extent to which management methods and procedures are developed;	• the proportion of facilities for primary health that have supplies such as essential medicines for MNS disorders.	
• the presence of an official policy, programme, or plan for mental health;		
• a specified budget for mental health as a proportion of the total health budget; and		
• the proportion of the total expenditure for mental health that is spent on community-based services.		

**Health Status**		

**Impacts**		
• the prevalence and burden (disability adjusted life years – DALYs) of mental and neurological disorders; and		
• deaths from suicide and the rate of self-inflicted injuries.		

The Lancet call for action to scale up mental health services [15] noted the importance of monitoring progress in achieving the objectives of the call and proposed a set of core and secondary development targets and indicators of progress (Table 2) while acknowledging that no consensus yet exists about which mental health indicators should be used for such monitoring. The indicators proposed in the call for action will enable monitoring of attainment of targets related to scaling up the coverage of basic, evidence-based services for mental disorders, and will enable comparison across countries. The 11 indicators selected (5 core and 6 secondary) address four important overarching goals: (1) sufficient planning and investment for mental health care; (2) a sufficient workforce to provide mental health services; (3) consistency of mental health care inputs and processes with best practice and human rights protection; and (4) improved outcomes for people with mental disorders.

**Table 2:** Lancet call for action – selected mental health targets, with core and secondary indicators [15]

**Core targets and indicators**	
Ensure that national and regional health plans pay sufficient attention to mental health	1: Presence of official policy, programs, or plans for mental health, either including or accompanied by a policy on child and adolescent mental health
Invest more in mental health care	2: Specified budget for mental health as a proportion of total health budget
Increase trained staff to provide mental health care	3: Mental health and related professionals per 100,000 population
Make basic pharmacological treatments available in primary care	4: Proportion of primary health-care clinics in which a physician or an equivalent health worker is available, and at least one psychotropic medicine of each therapeutic category (antipsychotic, antidepressant, mood stabiliser, anxiolytic, and antiepileptic) is available in the facility or in a nearby pharmacy all year long
Increase the treatment coverage for people with schizophrenia	5: People treated each year for schizophrenia as a proportion of the total estimated annual prevalence of schizophrenia

**Secondary targets and indicators**	

Balance expenditure in hospital and community services	6: Proportion of total mental health expenditure spent on community based services, including primary and general health-care services
Provide adequate basic training in mental health	7: Proportion of the aggregate total training time in basic medical and nursing training degree courses devoted to mental health
Distribute staff equitably between urban and rural areas	8: Proportion of psychiatrists nationally who work in mental health facilities that are based in or near the largest cities
Ensure least restrictive practice	9: Involuntary admissions as a proportion of all annual admissions
Protect the human rights of people with mental disorder	10: Presence of a national body that monitors and protects the human rights of people with mental disorders, and issues reports at least every year
Reduce the suicide	11: Deaths by suicide and self-inflicted injury rate

## The Observatory

The International Observatory on Mental Health Systems (IOMHS), an initiative of the Centre for International Mental Health, University of Melbourne, has been established in support of the WHO Mental Health Gap Action Programme and the objectives of the Movement for Global Mental Health. IOMHS is modelled on the successful European Observatory on Health Systems and Policies [16], which has been operating for a decade.

The overarching objective of IOMHS is to build capacity to measure and to track mental health system performance [17] in participating countries at national and sub-national (provincial and district) levels. Most important scaling up activities occur at local levels, while national level data are difficult to interpret and often hide as much as they reveal. For example, national level information on the mental health system in Indonesia or Sri Lanka will not reveal the substantial progress that has been made in developing community-based mental health services in some districts, or whether these developments are applicable and feasible in other parts of these two countries. Developing feasible methods to track progress in mental health system performance at district and provincial levels, and progress in disseminating new models of service delivery, is particularly important.

IOMHS will focus its attention particularly on low and middle-income countries that are active in implementing mental health service scaling up activities. The information that is produced by the Observatory will strengthen political commitment to scaling up of services and investment in mental health, inform the development of policies and plans, and will be used to strengthen human resources. It will also serve to protect and enhance the human rights of people with mental illness.

The work program of IOMHS will depend on the establishment of partnerships with governments, universities, international and local NGOs and other partner organisations that will collect, analyse and apply high quality information for mental health system reform and development. An outcome of such a collaborative work program will be the establishment of sustained and productive collaborations between mental health policy makers, academics, practitioners, NGOs, and civil society organisations, and improved capacity to analyse trends in health care reform. An explicit goal is to build the strengths of partner organisations and networks to provide evidence-based advice to national and sub-national policy makers, service planners and implementers.

**Figure 2:**
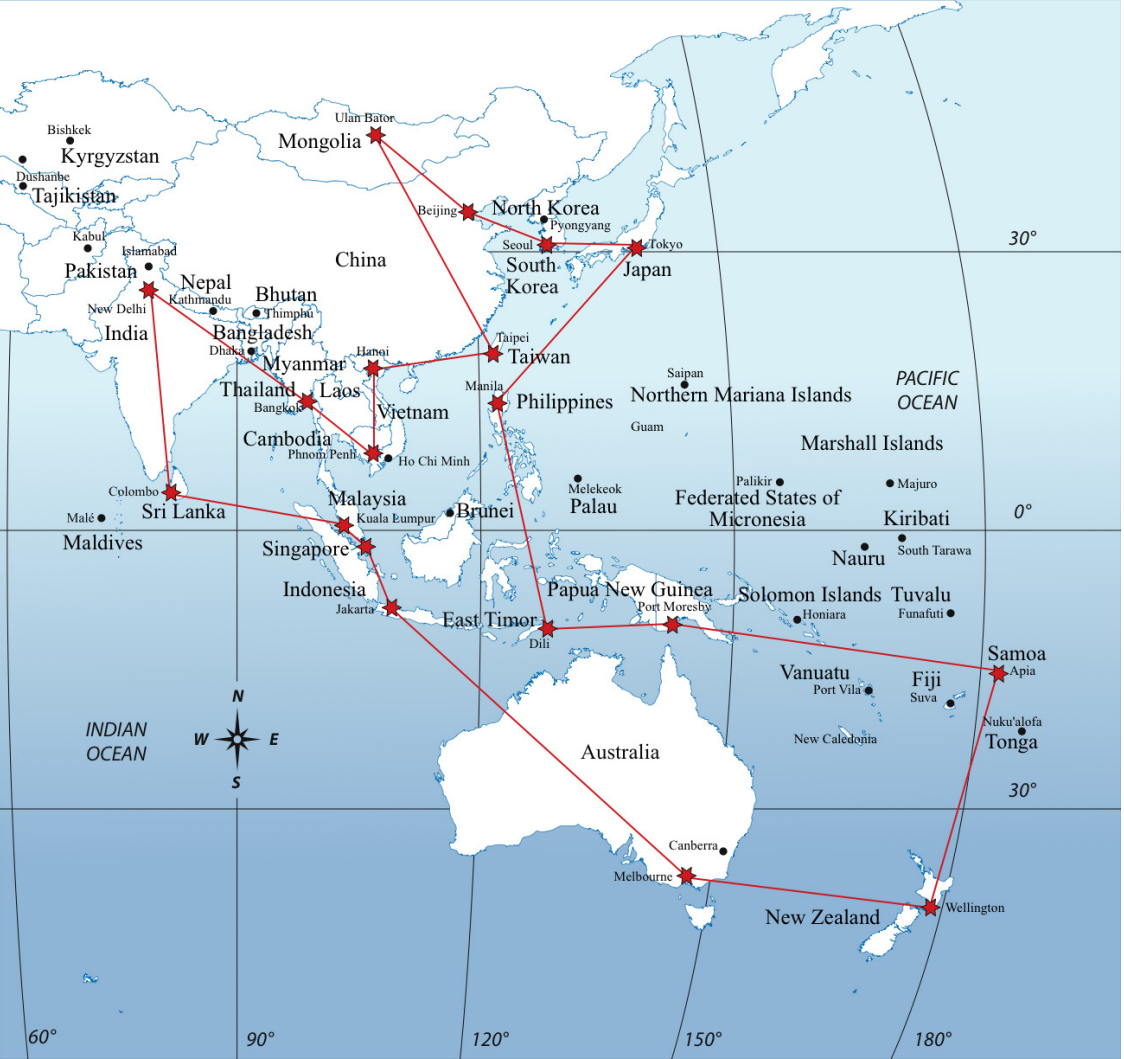
**International Observatory on Mental Health Systems, Asia Pacific**.

The Observatory will commence its program of work in Asia and the Pacific (Figure 2), building on the platform established by the International Mental Health Leadership Program [18] which has more than 100 alumni in 18 countries and territories in the Asia Pacific region.

### Initial IOMHS projects

In all world regions neuropsychiatric conditions are the most important causes of disability, accounting for over 37% of years lived with disability (YLDs) among those aged 15 years and over [19]. The contribution of neuropsychiatric conditions to global burden of disease is greatly under-estimated when this is measured by mortality alone [20,21]. In burden of disease projections to 2030 [22] the three leading causes of burden of disease are expected to be HIV/AIDS, depression and ischaemic heart disease, with road traffic accidents being either third or fourth depending on assumptions made in the projections. A key goal of scaling up coverage and accessibility of mental health services is to reduce the often severe disability that is so closely associated with mental illness [9,11,23,24]. This will mean that the packages of treatment and care that are developed as part of scaling up activities must go well beyond clinical treatment of mental disorders to include rehabilitation programs, education, housing and employment programs, and advocacy and human rights programs. IOMHS programs will be disability inclusive and will focus on work that improves our understanding of disability and mental health. Attention to disability will be a core feature of IOMHS programs that track the impact of scaling up activities.

As part of the Lancet series on global mental health a research priority-setting exercise was conducted to identify global mental health research priorities [15]. The method used was developed by the The Child Health and Nutrition Research Initiative [25]. Research priorities were identified for four key areas: 1) depressive disorders, anxiety disorders, and other common mental disorders; 2) alcohol-use and other substance-abuse disorders; 3) child and adolescent mental disorders; and 4) psychotic disorders. For each area the top five priorities were reported [15]. Mental health systems research constituted a substantial proportion of the top priorities in each of the four areas. These health systems research priorities will serve as a guide to the development of the IOMHS research program. The CHNRI method for establishing research priorities [25] will be used to expand on the mental health systems research priorities identified in the Lancet call for action [15] and to develop a consensus mental health systems research agenda for Asia and the Pacific. Such an agenda will be useful in fostering coherence and comparability of mental health systems research being carried out in the Asia Pacific region and will assist researchers to generate funds to carry out high priority research.

There is, at present, no agreed method for classifying mental health systems or for systematically comparing mental health systems across countries, or in any one country over time. This is despite the clear benefits that would be gained from having the capacity to carry out such systematic comparisons [26]. IOMHS will carry out work to develop a method that can be used to rationally classify mental health systems at national and sub-national (provincial and district) levels.

The WHO Mental Health Gap Action Program [5] and the Lancet call for action [15] have identified a number of targets and indicators for mental health service scaling up activities (Tables 1 and 2). The indicators identified are almost all measures of input and process that can be derived from existing national data collections. These measures are important but not sufficient to track progress of scaling up activities. There is a need to develop simple and robust measures of mental health system quality, and feasible methods of data collection, that will enable tracking of outcomes and impacts of scaling up activities. Most such activities are undertaken at district or provincial (i.e. sub-national) levels, activities that will almost certainly not be reflected in national level data collections. IOMHS will develop and trial a Mental Health Systems Index that can be used to track progress of scaling up activities at sub-national (provincial and district) levels.

It is currently not possible to accurately estimate levels of investment in mental health in most low and middle-income countries. It is not possible to significantly improve mental health system performance without increasing levels of investment in mental health services [27]. Nor is it possible to make sensible investment decisions about mental health systems in the absence reliable mental health economic data as part of national health accounts [28]. IOMHS will investigate and track levels of national investment in mental health services in countries in Asia and the Pacific.

### Secretariat and country programs

The *IOMHS *Secretariat is located in the Centre for International Mental Health, University of Melbourne. An International Steering Committee will be responsible for *IOMHS *policy and strategic directions and a Management Committee will be responsible for the operations of the program of work. As agreements are reached with governments, universities, and other partner organisations, and funds are obtained, it is anticipated that *IOMHS *country programs will be progressively established, led by a Country Research Director supported by research and administrative staff. The *IOMHS *Secretariat and the Country Programs will form a powerful mental health systems research and development network, capable of producing high quality evidence that will support the scaling up of mental health services in low and middle-income countries.

### Information dissemination

IOMHS is committed to making all publications open access and freely available at no cost. *International Journal of Mental Health Systems *[29] will be the primary means of dissemination of Observatory research. The Journal will publish research papers, reviews, case reports of mental health services, commentaries and policy briefs. Country experts will be invited to write mental health system country profiles, with the support of IOMHS research directors and staff. These country-based reports will provide detailed descriptions of national, provincial and district mental health systems, and policy and service development initiatives in progress. In order to facilitate comparisons between countries, and to track progress over time, the profiles will be based on a common report format. Mental health system country profiles will provide information to support policy makers and analysts in the development of mental health systems. Research reports and country profiles will be systematically translated into policy briefs, succinct summaries of policy and practice lessons written by international experts specifically for busy policy makers.

## Summary

The International Observatory on Mental Health Systems will be a mental health research and development network that will monitor and evaluate mental health system performance in low and middle-income countries. In order to do this work successfully the Observatory will rely on the establishment of robust partnerships between policymakers, service implementers, academics, practitioners, bilateral and multilateral development agencies, local and international NGOs, and funding agencies. Key goals will be to strengthen capability for monitoring and evaluation in low and middle-income countries, develop the necessary methods and feasible indicators for this purpose, and to focus attention at sub-national – provincial and district – levels, where scaling up activities mostly occur, as well as on national level data collections.

## Competing interests

The author declares that they have no competing interests.
